# Histological diagnosis of polyploidy discriminates an aggressive subset of hepatocellular carcinomas with poor prognosis

**DOI:** 10.1038/s41416-023-02408-6

**Published:** 2023-09-15

**Authors:** Takanori Matsuura, Yoshihide Ueda, Yoshiyuki Harada, Kazuki Hayashi, Kisara Horisaka, Yoshihiko Yano, Shinichi So, Masahiro Kido, Takumi Fukumoto, Yuzo Kodama, Eiji Hara, Tomonori Matsumoto

**Affiliations:** 1 Department of Molecular Microbiology, Research Institute for Microbial Diseases, Osaka University, Suita, Japan; 2 Division of Gastroenterology, Department of Internal Medicine, Kobe University Graduate School of Medicine, Kobe, Japan; 3 Division of Hepato-Biliary-Pancreatic Surgery, Department of Surgery, Kobe University Graduate School of Medicine, Kobe, Japan

**Keywords:** Liver cancer, Aneuploidy, Hepatocellular carcinoma

## Abstract

**Background:**

Although genome duplication, or polyploidization, is believed to drive cancer evolution and affect tumor features, its significance in hepatocellular carcinoma (HCC) is unclear. We aimed to determine the characteristics of polyploid HCCs by evaluating chromosome duplication and to discover surrogate markers to discriminate polyploid HCCs.

**Methods:**

The ploidy in human HCC was assessed by fluorescence in situ hybridization for multiple chromosomes. Clinicopathological and expression features were compared between polyploid and near-diploid HCCs. Markers indicating polyploid HCC were explored by transcriptome analysis of cultured HCC cells.

**Results:**

Polyploidy was detected in 36% (20/56) of HCCs and discriminated an aggressive subset of HCC that typically showed high serum alpha-fetoprotein, poor differentiation, and poor prognosis compared to near-diploid HCCs. Molecular subtyping revealed that polyploid HCCs highly expressed alpha-fetoprotein but did not necessarily show progenitor features. Histological examination revealed abundant polyploid giant cancer cells (PGCCs) with a distinct appearance and frequent macrotrabecular-massive architecture in polyploid HCCs. Notably, the abundance of PGCCs and overexpression of ubiquitin-conjugating enzymes 2C indicated polyploidy in HCC and efficiently predicted poor prognosis in combination.

**Conclusions:**

Histological diagnosis of polyploidy using surrogate markers discriminates an aggressive subset of HCC, apart from known HCC subgroups, and predict poor prognosis in HCC.

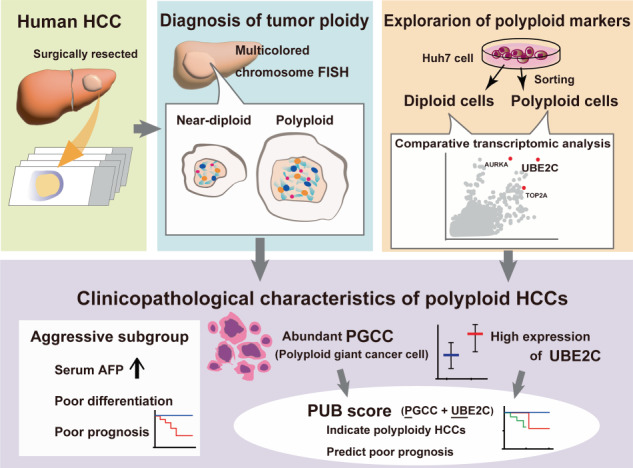

## Introduction

Accumulating evidence indicates that genome duplication or polyploidization is a crucial genetic trait of cancers. Pan-cancer genomic analysis has shown that a significant proportion of human solid tumors have experienced at least one round of polyploidization during their evolution [1, 2]. Polyploidization is considered to lead to chromosomal instability via frequent chromosomal missegregation during proliferation and by creating aneuploidy-permissive conditions [3–7], which in turn facilitates cancer evolution by inducing genetic diversity [5, 6]. In fact, polyploidization is estimated to occur prior to most copy number changes [8] and to promote metastasis in several cancer types such as pancreatic adenocarcinoma [2, 9].

Given that polyploidization is implicated in tumor evolution and shapes the characteristic landscape of cancer genomes, polyploid cancers may exhibit distinct features in their clinicopathological profiles. Some studies with massively parallel sequencing technologies have robustly evaluated genome duplications in tumors and demonstrated the general landscape of polyploid cancers [1, 2, 8, 10]. A pan-cancer study on human polyploid tumors, using bulk RNA sequencing, recently indicated that they overexpress genes important for cellular proliferation and mitotic spindle formation [1]. Polyploid cancers have also been suggested to be negatively correlated with the presence of tumor-infiltrating leukocytes [1]. However, details about the clinicopathological and expressional features of polyploid tumors have been largely unknown due in part to the difficulty of evaluating polyploidy in pathological specimens. Uncovering characteristics that represent polyploid tumors would enhance the possible utility of discriminating ploidy status of tumors in their clinical management.

Hepatocellular carcinoma (HCC) is the most common primary malignancy of the liver. Notably, polyploidization of hepatocytes has been implicated in chronic liver damage and cancer development in human and rodent studies [11–14]. In the past, flow cytometric analysis of human HCC specimens showed that HCCs were mostly diploid [15, 16]. In contrast, genomic analysis suggested that ~34% of HCCs have undergone polyploidization [1]. A recent study in which nuclear ploidies were inferred by image cytometric analysis of cellular nuclear sizes in HCC tissues also suggested that a subset of HCCs is predominantly composed of polyploid cells [17]. Although these studies have shown the significance of polyploidy in HCCs [1, 17], the details are conflicting, and the correlations with polyploidy and the molecular subgroups known in HCCs are unclear.

In this study, we assessed the ploidy status of human HCCs by directly evaluating multiple chromosome duplications using multicolored fluorescence in situ hybridization (FISH) and showed that approximately one-third of HCCs were predominantly polyploid. Polyploidy in HCC discriminated an aggressive subset of HCC with characteristic histology and poor prognosis, apart from the known molecular HCC subgroups. Exploration of surrogate markers revealed that the abundance of polyploid giant cancer cells (PGCCs) and overexpression of ubiquitin-conjugating enzyme E2 C (UBE2C) served as an indicator of polyploidy in HCC and efficiently predicted poor prognosis in combination. The diagnosis of polyploidy using pathological sections is proposed to serve as a novel prognostic marker for HCC.

## Materials and methods

### Clinical HCC specimens

A total of 56 HCC tissues were obtained from patients who underwent hepatectomy for HCC between 2017 and 2021 at Kobe University Hospital, Kobe, Japan. Clinical data, including age, sex, and etiology of HCC, were obtained from the patient’s medical records, and FFPE samples were used for analysis. This study was performed in accordance with the Declaration of Helsinki and approved by the ethics committee of Kobe University School of Medicine. Opt-out consent was obtained because the study was retrospective.

### Histology, immunostaining

FFPE tissues were cut into 5 µm sections and subjected to hematoxylin (Sakura 3G, Sakura Finetek, Japan) and eosin (1% Eosin Y solution, Muto Pure Chemicals, Tokyo, Japan) or immunostaining. Immunohistochemistry was performed using primary antibodies against UBE2C (1:500, ab252940, Abcam), TOP2A (1:500, #12286, Cell Signaling Technology), AURKA (1:500, #91590, Cell Signaling Technology), HNF4a (1:200, #3113, Cell Signaling Technology), CD3 (no dilution, 413591, Nichirei. Bio), AFP (no dilution, 418291, Nichirei Bio), SALL4 (1:150, #101147, Santa Cruz), EPCAM (1:300, #93790, Cell Signaling Technology), pHH3 (1:200, #9701, Cell Signaling Technology), p21 (1:100, #2947, Cell Signaling Technology), IL6 (1:1000, ab9324, Abcam), Ki-67 (1:300, ab15580, Abcam), and HNF4a (1:200, #3113, Cell Signaling Technology), and secondary antibodies including anti-rabbit (MP-7401, Vector Laboratories, Burlingame, CA), anti-mouse (MP-7402, Vector Laboratories), and Alexa Fluor 488-conjugated anti-rabbit (1:200, Jackson ImmunoResearch). The nuclei were counterstained with hematoxylin or DAPI (F10347, Thermo Fisher Scientific). All slides were stained with their primary antibody at 4 °C overnight. HE- and immunohistochemically stained slides were photographed using an Olympus DP-27 microscope with the Olympus cellSens microscopy software (Olympus, Tokyo, Japan). Fluorescence images were analyzed using a fluorescence microscope (BZ-X710, Keyence, Osaka, Japan) and BZ-X analyzer software (Keyence).

### Chromosome FISH and identification of polyploid HCCs

The 5 μm FFPE sections were used for multicolored chromosome FISH. FISH was performed using chromosome enumeration DNA FISH probes that were specific to the pericentromeric regions of chromosomes 7 (#6J36-77), 11 (#6J54-21), and 16 (#5J10-26) according to the manufacturer’s instructions (Abbott Laboratories Ltd, IL). The slides were denatured at 72 °C for 6 min, hybridized at 37 °C overnight, and counterstained with DAPI. The FISH slides were examined at 100× magnification under a fluorescence microscope (BZ-X710, Keyence). The number of signals for chromosomes 7, 11, and 16 in the tumor nucleus was determined as the chromosome copy number of the nucleus, and their predominant numbers in all fields examined was determined as the chromosome copy numbers of the tumor. The average of the three chromosome copy numbers were calculated to assess HCC ploidy.

### PUB score

The PUB score was defined as follows: PUB score 0 indicates neither PGCC-abundant nor UBE2C-moderate/strong; PUB score 1 indicates either PGCC-abundant or UBE2C-moderate/strong; PUB score 2 indicates both PGCC-abundant and UBE2C-moderate/strong. PGCC abundance was defined by whether one or more PGCC were observed per 40× magnified field.

### Statistical analysis

Statistical analysis was performed using the one-sided Mann–Whitney *U* test, Student’s *t* test, chi-square test, linear regression modeling, or Kaplan–Meier survival analysis, using R software (version 4.1.2, R Foundation for Statistical Computing), Microsoft Office Excel (Microsoft), and Prism 9 (GraphPad Software, Inc.). In the comparison of DEGs detected by transcriptome analyses, *p* values were calculated by a hypergeometric distribution test using R software.

For further details regarding the materials and methods, please refer to supplementary materials and methods.

## Results

### FISH targeting for multiple chromosomes distinguished ploidy status in HCCs

**Fig. 1:**
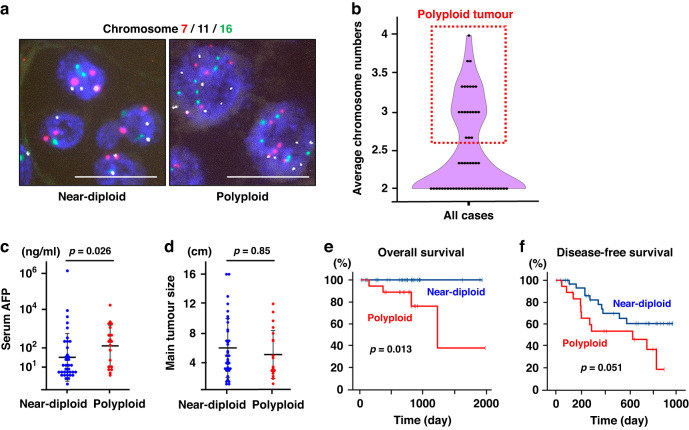
Determination and clinical features of polyploid HCCs. **a** Multicolored chromosome FISH. Red, white and green dots indicate signals detecting chromosome 7, 11, 16, respectively. DAPI is shown in blue. Scale bars, 20 μm. **b** A violin plot of average chromosome numbers in each HCC. The copy numbers of three chromosomes per tumor cell nucleus were examined by evaluating more than 200 tumor nuclei at 5 or more high-magnification fields, and their predominant values were determined in each tumor. The average values of three stained chromosomes were plotted, and HCCs with 2.5 or more copies of chromosomes in average were defined as polyploid based on the bimodal distribution. **c** Serum AFP levels. **d** The main tumor size. Mann-Whitney *U* test in (**c**) and (**d**). Kaplan-Meier curves of overall survival (**e**) and disease-free survival (**f**). Error bars indicate mean ± SD.

To assess genome duplications and their frequency in HCCs, formalin-fixed paraffin-embedded (FFPE) sections of 56 surgically treated HCCs were analyzed using FISH for chromosomes. Three chromosomes were simultaneously stained using multicolored FISH to examine the copy number of each chromosome per tumor cell nucleus (Figs. 1a, S1A), and the predominant copy numbers of the three chromosomes were determined in each tumor. The average of the predominant copy numbers was supposed to represent the ploidy of each tumor, and polyploidy was determined based on its characteristic bimodal distribution (Fig. 1b). In this criterion, the majority (>50%) of tumor cells in polyploid HCCs were composed of cells with polyploid nuclei by definition, and twenty HCCs were determined to be polyploid (35.7%). The average ploidy of these polyploid HCCs was estimated to be 3.14. Chromosome duplications (i.e., four copies of chromosomes) were exclusively observed in most polyploid HCCs, strongly suggesting a history of genome duplication (Fig. S1B).

To validate our assessment of polyploidy by chromosome FISH, the nuclear size and DAPI intensity of the HNF4α-positive/Ki-67 negative tumor cells were selectively evaluated by image cytometry (Fig. S1C). The integrated nuclear DAPI fluorescence, which proportionally reflects DNA content [18, 19], was significantly higher in the tumor cells of polyploid HCCs than in those of near-diploid tumors (Fig. S1D, E). The distribution of the integrated DAPI intensities verified that polyploid HCCs were predominantly composed of genome-amplified tumor cells, whereas the majority of tumor cells in near-diploid HCCs were diploid (Fig. S1D). Moreover, nuclear size, which is correlated with DNA amount [13, 17, 20], was significantly larger in polyploid HCCs than in near-diploid HCCs (Fig. S1F). These tumor cell nuclei findings clearly confirmed that the tumor ploidy status was appropriately determined.

### Polyploidy discriminated HCCs with an aggressive clinical feature

We first explored and compared the clinicopathological features of polyploid HCCs and near-diploid HCCs. There were no significant differences in patient age, sex, body mass index, or performance status between the two groups (Table 1). While fatty liver diseases, including alcoholic and non-alcoholic steatohepatitis, were likely to be detected as the etiology of polyploid HCCs, a trend toward hepatitis virus infection was observed in near-diploid HCCs (Table 1). Notably, although the tumor size and stages were comparable between polyploid and near-diploid HCCs, the serum alpha-fetoprotein (AFP) level was significantly higher in polyploid HCCs (Fig. 1c, d, Table 1). Furthermore, polyploidy was associated with significantly worse overall survival (*p* < 0.01) and a trend toward worse disease-free survival after surgery (*p* = 0.05, Fig. 1e, f). The negative impact of polyploidy on the prognosis of HCCs was also confirmed in The Cancer Genome Atlas data set (Fig. S2). These findings indicate that polyploidy can be used to discriminate HCCs with aggressive clinical features.

**Table 1:** Clinicopathological information of 56 HCC patients analyzed in the present study.

	Near-diploid	Polyploid	Significance
Case	36	20	
Age (median ± SD)	72.5 ± 9.6	72 ± 14.0	n.s.^a^
Sex (male/female)	24/12	18/2	*p* = 0.06^b^
Body mass index (kg/m^2^)	22.6 ± 3.0	22.6 ± 4.8	n.s.^a^
Performance status (PS0/1/2)	31/5/0	17/1/1	
Etiology (HBV/HCV/HBV + HCV/Alcohol/ NASH/PBC + AIH/unknown)	13/7/4/3/3/2/4	4/3/2/6/3/1/1	
Total bilirubin (mg/dL)	0.8 ± 0.4	0.6 ± 0.3	n.s.^a^
AFP (ng/mL)	9.8 ± 214885.9	193 ± 234358.5	*p* = 0.026^a^
DCP (mAU/mL)	316.5 ± 11634.9	405 ± 31599.4	n.s.^a^
Inflammation (A0/1/2/3/unknown)	4/8/3/0/21	1/1/2/0/16	
Fibrosis (F0-1/2/3-4)	11/16/8	5/7/8	n.s.^b,c^
Tumor size	4.3 ± 4.2	4.1 ± 13.3	n.s.^a^
BCLC stage (A/B/C)	16/16/4	11/6/3	
Tumor classification (Simple nodular/Contiguous multinodular/Others)	24/8/2	12/6/2	
Differentiation (Well/Moderately/Poorly)	3/28/5	0/9/11	*p* = 0.002^b,d^
Pathological structure (Microtrabecular/Macrotrabecular/Compact/Pseudoglandular/Schirrhous/Unclassified)	20/3/5/4/2/2	2/7/8/1/0/2	*p* = 0.025^b,e^

^a^Wilcoxon-Mann-Whitney U test,

^b^Fisher’s exact test,

^c^F0-2 vs. F3-4,

^d^Poorly vs. well/moderately,

^e^Macrotrabecular-massive vs. the others.

### Polyploid HCCs were poorly differentiated and harbor polyploid giant cancer cells

**Fig. 2:**
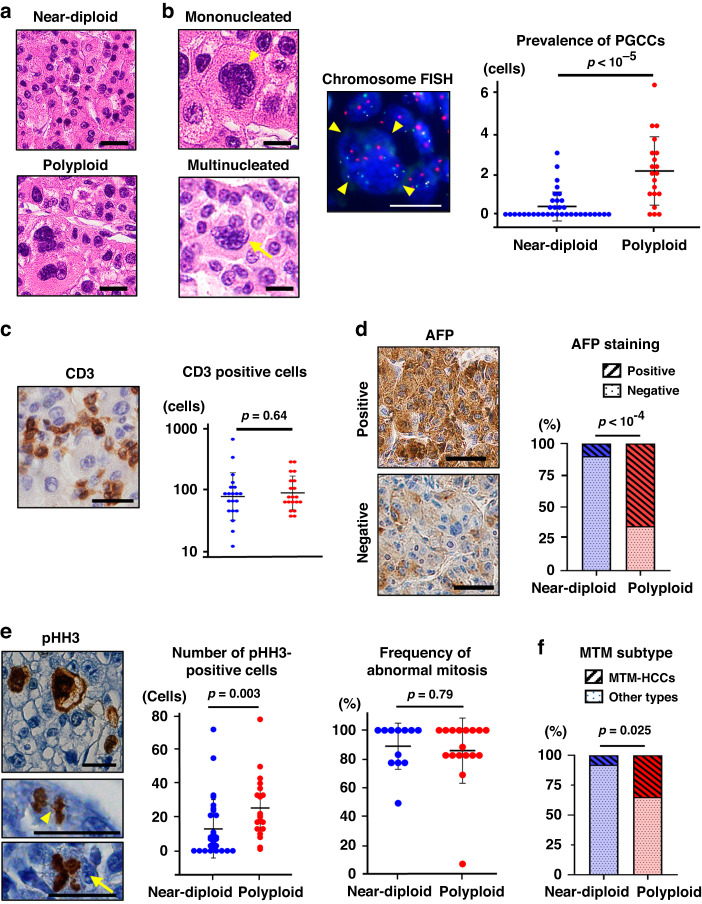
Histological features of polyploid HCCs. **a** HE staining of near-diploid and polyploid HCCs. **b** PGCCs in HCC. HE staining and chromosome FISH of PGCCs are shown. Arrowheads indicate mononucleated PGCCs with an irregularly shaped, giant nucleus. An arrow indicates a multinucleated PGCC. **c** Numbers of CD3 positive cells. A microscopic image of CD3 staining is also shown. Immunostaining for AFP (**d**) and pHH3 (**e**). A chromosome bridge (an arrowhead) and multipolar mitosis (an arrow) are considered as abnormal mitosis. **f** Proportion of macrotrabecular-massive HCCs (MTM-HCCs). In (**b**), (**c**), and (**e**), numbers of PGCCs, CD3-positive, and pHH3-positive cells per 40× magnified field were calculated from 5 or more fields in each tumor, respectively. Error bars indicate mean ± SD. Scale bar, 50 μm except for (**b**, FISH), 20 μm. Student’s t-test in (**b**), (**c**) and (**e**), and chi-square test in (**d**) and (**f**).

Next, we sought to characterize the histological features of the polyploid and near-diploid HCCs. Notably, polyploid HCCs were significantly poorly differentiated compared to near-diploid tumors (Table 1). Consistent with the image cytometry results (Fig. S1E), the polyploid HCCs frequently contained large nuclei (Fig. 2a). Intriguingly, tumor cells with prominently large nuclei and those with profound multinucleation were occasionally detected in the polyploid HCCs (Fig. 2b). Multicolored chromosome FISH confirmed that cells with large nuclei were highly polyploid (Fig. 2b). These distinct cancer cells can be recognized as PGCCs [21, 22].

**Fig. 3:**
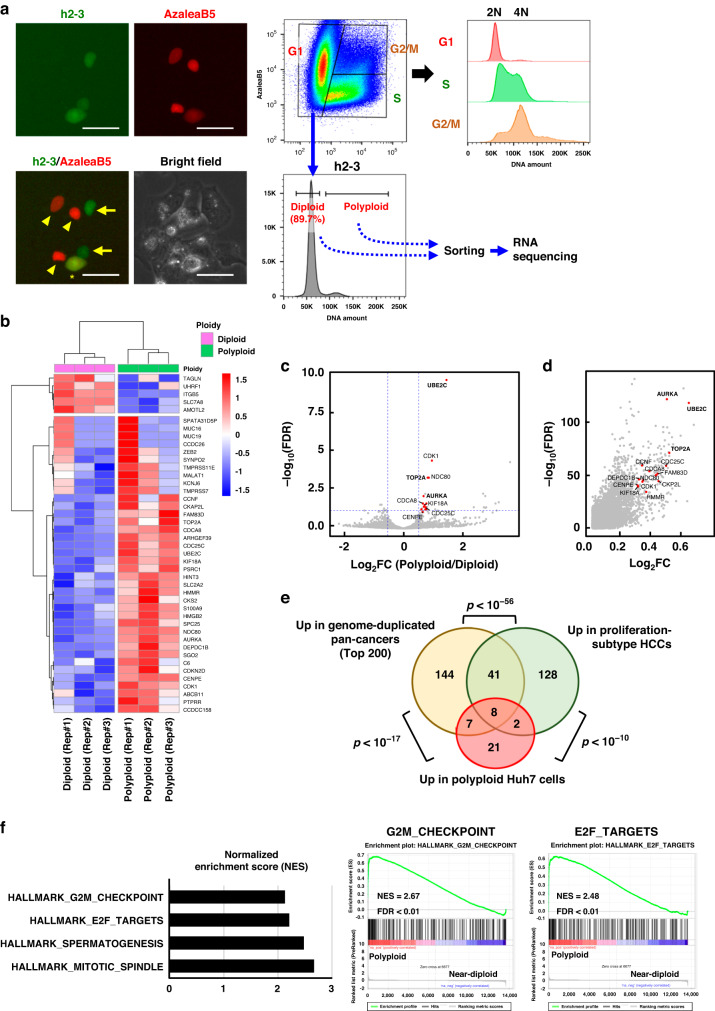
Expressional features of polyploid HCCs. **a** Fluorescent images and FACS plots of Huh7-Fucci cells. AzaleaB5 (red)-single positive (arrowheads), h2–3 (green)-single positive (arrows), and AzaleaB5/h2-3 double-positive (an asterisk) cells represent those in G1, S, and G2/M phases, respectively. Scale bar, 50 μm. Note that cells in S and G2/M phases harbor increased DNA due to the genome replication during S phase, whereas G1 cells exhibit sharp modal distribution with a fraction of polyploid cells. **b** Heatmap of RNA sequencing data. DEGs between diploid and polyploid cells are shown. **c** Volcano plot comparing polyploid and diploid Huh7-Fucci cells. The dashed lines indicate FDR = 0.05 and |log_2_(FC)| = 0.5 thresholds. **d** Volcano plot of upregulated DEGs in polyploid tumors shown by pan-cancer bulk RNA sequencing [1]. In (**c**) and (**d**), genes commonly upregulated in polyploid Huh7-Fucci cells and polyploid tumors are plotted in red, and some of their names are shown. **e** Venn diagram of upregulated DEGs in polyploid HCC cells (this study), polyploid tumors [1], and proliferation-subtype HCCs [34]. Genes with top 200 smallest FDR values were considered in polyploid tumors. **f** Gene set enrichment analysis of the transcriptomes comparing diploid and polyploid Huh7-Fucci cells. Rep Replicate, FC Fold change, FDR False discovery rate, NES Normalized enrichment score.

We defined PGCCs as a cell whose nucleus was at least three times larger than that of a regular cancer cell [23, 24] and examined the prevalence of PGCCs in tumors. Notably, PGCCs were predominantly detected in polyploid HCCs than in near-diploid tumors (Fig. 3d). In contrast, the prevalence of PGCCs did not correlate with the tumor differentiation grade and tumor size (Fig. S3A, B), suggesting that the existence of PGCCs was indicative of genome doubling in HCCs regardless of the tumor grade.

Previous studies have reported conflicting results on the correlation between cancer polyploidization and intratumoral infiltration of immune cells particularly among HCCs [1, 17, 25]. Interestingly, we evaluated tumor-infiltrating lymphocytes by immunostaining for the T-cell marker CD3 in our case series, which revealed a comparable number of intratumoral lymphocytes between polyploid and near-diploid HCCs (Fig. 2c). The inflammation and fibrosis status of the background non-tumorous liver tissues were also comparable, irrespective of polyploidy in HCCs (Table 1). These findings suggest that polyploidy in HCC is correlated with poor tumor differentiation and the abundance of PGCCs but not with a difference in the number of tumor-infiltrating lymphocytes.

### Polyploid HCCs exhibit an aggressive phenotype, different from known molecular HCC subgroups

The higher serum AFP levels and poorer prognosis in polyploid HCCs prompted us to investigate their biological properties in more detail [26]. Immunohistochemical analysis of AFP in HCC tissues demonstrated a marked difference in positivity by ploidy status (Fig. 2d). The frequencies of tumor cells positive for pHH3, a mitotic marker, revealed significantly more aggressive proliferation of cancer cells in polyploid HCCs than in near-diploid HCCs (Fig. 2e). The frequency of abnormal mitotic structures including chromosome bridges and multipolar mitoses was quite high (~90%), irrespective of the ploidy status (Fig. 2e).

To examine whether polyploid HCCs exhibit progenitor features that define a subgroup of HCC with aggressive phenotypes [26–28], the immunopositivity of EpCAM, a marker for ductal transdifferentiation of hepatocytes, was further evaluated. Although polyploid HCCs overexpressed EpCAM more frequently than near-diploid HCCs (20% vs. 10%), the difference was not significant and the overall frequency of EpCAM-positive HCCs was limited (15%, 6/40), consistent with previous studies (Fig. S4A) [29, 30]. Moreover, the positivity of SALL4, which is a putative key transcription factor of progenitor-subtype HCC and reportedly exhibits higher positivity in HCCs than EpCAM [31, 32], was comparable between polyploid and near-diploid HCCs (43.5% vs. 40%, Fig. S4B). Importantly, the positivity of AFP, EpCAM, and SALL4 immunostaining showed an insignificant impact on the prognosis of HCCs compared to the ploidy status (Fig. S4C–E).

Macrotrabecular-massive HCCs are another subgroup of HCC with an aggressive phenotype [26, 29]. We examined the architectural growth patterns of the histology in detail (Fig. S5) and found that macrotrabecular-massive HCCs were significantly predominant in polyploid HCC (Fig. 2f). However, a considerable proportion of polyploid HCCs (65%, 13/20) showed histological architecture different from macrotrabecular-massive HCC (Fig. 2f). These findings indicate that polyploidy discriminates a subset of HCCs with an aggressive phenotype and poor prognosis, different from the previously known molecular and morphological HCC subgroups [26].

### Comparative transcriptomic analysis of human HCC cells revealed gene sets that were highly expressed in polyploid cancer cells

To explore the characteristics of polyploid HCC cells and the markers that represent them, we analyzed the transcriptomes of Huh7 human hepatoma cell lines with different ploidies. DNA content alone cannot be used to distinguish diploid from polyploid cells because diploid cells can have duplicated DNA content during late S, G2, and M phases. Therefore, the fluorescent ubiquitination-based cell cycle indicator (Fucci) system [33] was introduced into Huh7 cells (Fig. 3a). Flow cytometric analysis of the DNA content in Huh7-Fucci cells clearly showed that approximately 10% of Huh7 cells in the G1 phase were polyploid (Fig. 3a). These polyploid Huh7 cells in the G1 phase and diploid G1 cells were collected by FACS, and the extracted RNAs were analyzed by RNA sequencing (Figs. 3a and S6).

Paired comparisons between diploid and polyploid cells identified significant differentially expressed genes (DEGs) between diploid and polyploid cells (Fig. 3b, c, Tables S1 and S2). Interestingly, there was a strong correlation (*p* < 10^−17^) between 39 upregulated DEGs in polyploid Huh7 cells and genes overexpressed in human genome-doubled tumors shown by pan-cancer bulk RNA sequencing analysis (Fig. 3d, e) [1]. In addition, these upregulated gene lists were quite similar to those upregulated in proliferation-subtype HCC [34], supporting the notion that polyploid HCCs represent a subset of HCCs exhibiting aggressive features with abundant proliferation.

Furthermore, gene set enrichment analysis of our transcriptome data derived from Huh7 cells revealed that gene sets associated with the G2M checkpoint, targets of the transcription factor E2F, and mitotic spindles were significantly enriched in polyploid Huh7 cells (Fig. 3f). All of these gene sets were also enriched in a previous bulk transcriptome analysis of polyploid pan-cancers [1]. The agreement between our data and pan-cancer data strongly suggested the expressional features of polyploid cancer cells, such as the upregulation of genes related to chromosome segregation and mitosis progression.

### Enhanced expression of UBE2C and AURKA is a surrogate marker for polyploid HCCs and is strongly correlated with poor prognosis

We further investigated whether the DEGs upregulated in polyploid cancer cells can serve as surrogate markers for polyploidy in HCCs. We focused on three genes that were most significantly upregulated in polyploid Huh7 cells and in genome-duplicated cancers. The three genes were *UBE2C*, aurora kinase A (*AURKA*), and DNA topoisomerase II alpha (*TOP2A*) (Fig. 3c, d). Examination using another human epithelial cell line, RPE1, which expresses Fucci, demonstrated significant or likely upregulation of the transcriptional expression of these genes in polyploid cells (Fig. S7).

**Fig. 4:**
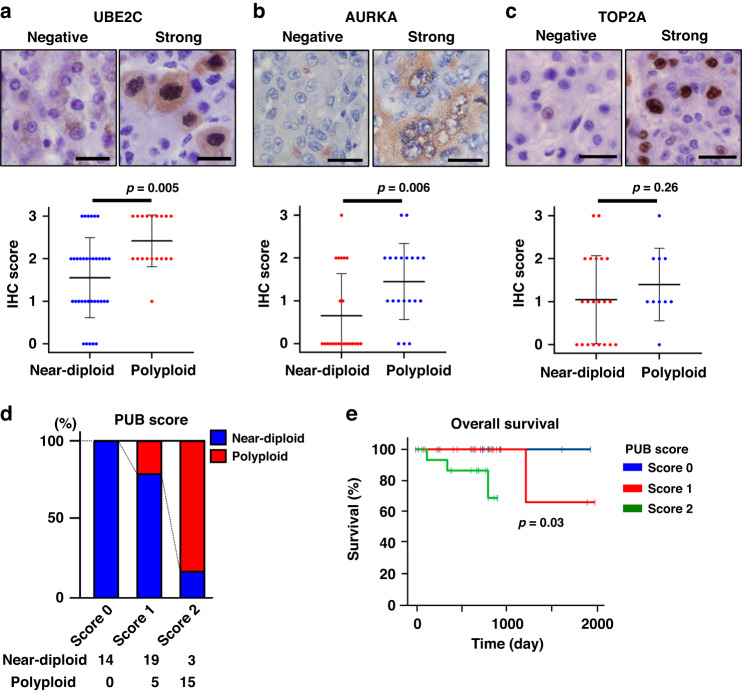
Expressions of UBE2C, AURKA, and TOP2A in polyploid HCCs. Immunostaining for UBE2C (**a**), AURKA (**b**), and TOP2A (**c**) in near-diploid and polyploid HCCs. For the scoring of staining intensity, please see Supplementary Fig. 7. Scale bar, 50 μm. **d** Prevalence of near-diploid and polyploid HCCs stratified by PUB scores. **e** Kaplan-Meier curve of overall survival stratified by PUB scores. For PUB scores, please see supplementary materials and methods. Student’s *t* test in (**a**), (**b**) and (**c**).

UBE2C is an E2 ubiquitin-conjugating enzyme specifically involved in the anaphase-promoting complex/cyclosome [35]. The expression of UBE2C proteins was variable among the HCCs (Figs. 4a and S8) and significantly higher in polyploid HCCs than in near-diploid HCCs (Fig. 4a). Notably, almost all polyploid HCCs showed moderate or strong UBE2C expression (Figs. 4a and S8). AURKA plays a vital role in regulating centrosome maturation and spindle formation during mitosis [36]. The expression of the AURKA protein was significantly higher in polyploid HCCs than in near-diploid HCCs (Fig. 4b). In contrast, TOP2A, which regulates sister chromosome segregation as a core component of mitotic chromosomes [37, 38], was expressed at similar levels regardless of the ploidy status (Fig. 4c). These findings indicate that some regulators of chromosome segregation and mitosis, including UBE2C and AURKA, are highly expressed at the protein level in polyploid HCCs.

Among the several features of polyploid HCCs found here, the abundance of PGCCs (Fig. 2b) and the overexpression of UBE2C (Fig. 4a) exhibited the highest sensitivity for detecting polyploidy in HCCs (Table S3). Thus, we further examined whether these two tests could predict ploidy status in combination. Notably, most tumors with abundant PGCCs and UBE2C overexpression were polyploid, whereas all tumors that were neither PGCC-abundant nor UBE2C-moderate/strong were near-diploid (Fig. 4d). The score based on these two elements (PGCC/UBE2C; PUB score) effectively predicted the ploidy status in HCCs: A PUB score of 2 indicating PGCC-abundant and UBE2C-moderate/high predicted polyploidy in HCCs with a sensitivity of 75% and a specificity of 92%. Moreover, tumors with a PUB score of 2 showed a significantly poorer prognosis than the other tumors (Fig. 4e). The prognosis prediction by the PUB score was independent of the serum AFP levels (Fig. S9A) and was more efficient than serum AFP (Fig. S9B, C). These findings suggest that UBE2C expression and the abundance of PGCCs serve as surrogate markers indicative of polyploidy in HCCs and predict poor prognosis.

### PGCCs in HCCs exhibit heterogeneous features

Although PGCCs are prominent owing to their discriminative nuclear appearance, their biological characteristics have not been determined. Some studies have shown that PGCCs exhibit senescent or stem-like features, which is controversial [23, 39].

**Fig. 5:**
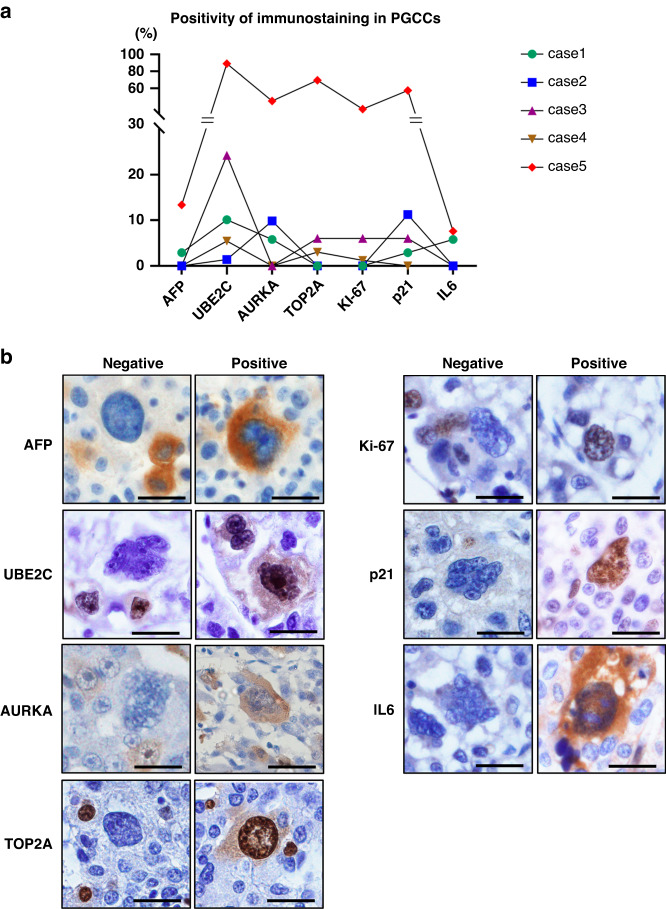
Expressional characteristics of PGCCs in HCC. **a** Positivity of immunostainings in PGCCs. HCC with abundant PGCCs (*n* = 5) were analyzed. **b** Immunostaining images of PGCCs. Scale bar, 50 μm.

We examined the expressional features of PGCCs by quantitative evaluation of immunopositivity using HCCs with abundant PGCCs. About 5–10% of PGCCs were positive for polyploid markers such as UBE2C and AURKA, while others were not (Fig. 5). The positivity rate was not consistent among the tumors examined, and PGCCs in one HCC were quite frequently (~80%) positive for UBE2C and AURKA (Fig. 5a). Senescence markers, such as p21 and IL6, also showed similar diverse positivity in PGCCs (Fig. 5). Moreover, some PGCCs were positive for the proliferation marker Ki-67 (Fig. 5), suggesting that senescence is not a universal feature of PGCCs, and that some PGCCs are proliferative. Although PGCCs in some cancers have been reported to exhibit stem cell features [21], PGCCs positive for the oncofetal protein AFP was relatively rare among polyploid HCCs (Fig. 5).

Taken together, these findings indicated that hepatocellular PGCCs that emerge without therapeutic interventions exhibit heterogeneous expressional features while they share a discriminative appearance such as huge and/or multinucleated nuclei.

## Discussion

Although most studies analyzing polyploidization in cancers have utilized massively parallel sequencing technologies to evaluate genome duplications [1, 2, 8, 10], they can only computationally infer allelic copy number profiles [40] and cannot directly demonstrate genome duplication. They also lose information regarding the morphological and expressional features of individual polyploid cancer cells. In the present study, polyploidy in cancer cells was directly demonstrated by multicolored chromosome FISH, and the ploidy status of HCCs was confirmed. The frequency of polyploid HCCs in the present study was consistent with that inferred by sequencing analysis (36% vs. 34%) [1], confirming that a significant proportion (approximately one-third) of HCCs were polyploid. Although flow cytometric analysis previously reported a lower frequency (<20%) of polyploid HCCs [15, 16], contamination with abundant diploid non-tumorous cells would distort the ploidy spectrum of samples and lead to the underestimation of polyploid HCCs. Even though chromosomal gain/loss and heterogeneity of tumor cells might affect the results of chromosome FISH, image cytometric evaluation of cancer nuclei (Fig. S1C–E) confirmed that polyploid HCCs assessed by chromosome FISH were composed of tumor cells with high DNA content.

Polyploid HCCs were significantly more poorly differentiated and had a poorer prognosis than near-diploid HCCs, which was consistent with a previous report where highly polyploid HCCs were inferred by nuclear sizes [17]. Considering the reported molecular subgroups of HCCs based on genomic, expressional, and/or histological features (reviewed in ref. [26]), polyploid HCCs are assumed to represent a high-proliferation subclass with a more aggressive phenotype, which exhibits poorly differentiated histology and high AFP serum levels [26, 34, 41]. Intriguingly, this high-proliferation subclass is closely related to chromosomal instability [26], coinciding with the notion that polyploidization encourages chromosomal instability [1, 10, 42]. In contrast, polyploid HCCs were not correlated with a “progenitor” or macrotrabecular-massive subtype, both of which are proposed to be classified among the high-proliferation HCC subclasses [29, 30]. Indeed, the prevalence of EpCAM-positive “progenitor”-subtype HCCs was comparable between polyploid (20%) and near-diploid (10%) HCCs (and reportedly 14–25% in all HCCs [29, 30, 43]) and was much less frequent than that of polyploid HCCs (~36%). Moreover, macrotrabecular-massive HCCs [29] were predominantly, but not exclusively, polyploid HCCs, and a considerable proportion of polyploid HCCs did not exhibit macrotrabecular-massive architecture (Fig. 2f). Polyploidy in HCCs represents highly proliferative HCCs without being restricted to known HCC subgroups.

The aggressive phenotype of polyploid HCCs, regardless of tumor grade, highlights the possible utility of using ploidy status as a predictive marker in HCCs. Some critical genetic alterations, such as *TP53* mutations and chromosomal instability, are known to affect tumor prognosis but are difficult to detect in clinical practice. We showed that the abundance of PGCCs and the expression of a surrogate marker, UBE2C (PUB score) could presume polyploidy in HCCs and predict poor prognosis, although not all the polyploid HCCs could be discriminated using these markers. Notably, the strong concordance between our transcriptomic screening using polyploid Huh7 cells and bulk RNA sequencing of genome-duplicated pan-cancers [1] suggests that the expression of *UBE2C* is robustly associated with polyploidy in cancers. While PGCCs characterized by a single giant nucleus or multinucleation have not been comprehensively analyzed in HCCs, they have been described in various types of cancers and suggested to facilitate chemoresistance of tumors [39, 44–47]. Notably, recent studies suggest that PGCCs acquire stemness, and regrowth from PGCCs via de-polyploidization leads to tumor evolution [48, 49]. Polyploidization and subsequent ploidy alterations have also been shown to promote liver cancer development in mice [12, 50]. PGCCs may not only serve as a marker but also contribute to tumor aggressiveness in polyploid HCCs. Prospective studies with large sample size are needed to investigate the significance of UBE2C expression and PGCCs in HCCs and whether they serve as an indicator of polyploidy and poor prognosis of HCCs.

Some questions related to genome duplication in HCCs remain unanswered. Conflicting results on tumor-infiltrating immunocytes in genome-duplicated cancers have been shown in HCCs ([17, 25] and the present study) and in pan-cancer analysis [1]. While Bou-Nader et al. demonstrated that HCCs with immune infiltration tended to consist of polyploid tumor cells, a recent study by Zhang et al. revealed a decrease in CD8-positive cytotoxic T cells and an increase in FoxP3-positive regulatory T cells in polyploid HCCs [17, 25]. In our current study, we found no correlation between the number of infiltrating CD3-positive T cells and the ploidy status of HCCs (Fig. 2c). These seemingly contradictory findings may be attributed to variations in the subsets of immune cells analyzed. Since liver cancers generally arise in inflamed livers, inflammation in the background liver might also have affected the immune ecosystem in HCCs regardless of ploidy status. Further investigation is warranted to elucidate the immunological characteristics and assess the efficiency of immunotherapy in polyploid HCC. The relationship between polyploidy and HCC etiology is also still unclear. In the present study, nonalcoholic and alcoholic steatohepatitis were more frequent in polyploid HCCs (Table 1). Among liver damages that can enhance hepatocyte polyploidization [51, 52], steatohepatitis is known to lead to hepatocyte polyploidization via excessive oxidative stress [13]. Further studies with larger sample sizes would answer these questions and uncover the implications of polyploidization in the clinical management of liver cancers.

Overall, we elucidated the clinicopathological features of polyploid HCCs and characterized them as a unique molecular HCC subgroup. Discrimination of polyploidy in HCCs using its surrogate marker would serve as a novel prognostic predictor, and polyploidy might be an innovative therapeutic target for an aggressive subset of HCCs. Given that polyploidization is thought to be a key evolutionary event in various types of tumors, polyploidy may be a promising general hallmark of cancer to diagnose and target aggressive malignancies.

### Supplementary information

Supplementary Text and figures

Supplementary Table1

Supplementary Table2

Supplementary Table3

## Data Availability

RNA sequencing data generated in this study have been deposited in the DDBJ Sequence Read Archive (https://www.ddbj.nig.ac.jp) under the accession number DRA014730. The authors declare that all other data are available upon request.

## References

[1] Quinton RJ, DiDomizio A, Vittoria MA, Kotýnková K, Ticas CJ, Patel S (2021). Whole-genome doubling confers unique genetic vulnerabilities on tumour cells. Nature..

[2] Bielski CM, Zehir A, Penson AV, Donoghue MTA, Chatila W, Armenia J (2018). Genome doubling shapes the evolution and prognosis of advanced cancers. Nat Genet.

[3] Taylor AM, Shih J, Ha G, Gao GF, Zhang X, Berger AC (2018). Genomic and functional approaches to understanding cancer aneuploidy. Cancer Cell.

[4] Van de Peer Y, Mizrachi E, Marchal K (2017). The evolutionary significance of polyploidy. Nat Rev Genet.

[5] Ben-David U, Amon A (2020). Context is everything: aneuploidy in cancer. Nat Rev Genet.

[6] Sansregret L, Vanhaesebroeck B, Swanton C (2018). Determinants and clinical implications of chromosomal instability in cancer. Nat Rev Clin Oncol.

[7] Wangsa D, Quintanilla I, Torabi K, Vila-Casadesús M, Ercilla A, Klus G (2018). Near-tetraploid cancer cells show chromosome instability triggered by replication stress and exhibit enhanced invasiveness. FASEB J.

[8] Gerstung M, Jolly C, Leshchiner I, Dentro SC, Gonzalez S, Rosebrock D (2020). The evolutionary history of 2,658 cancers. Nature..

[9] Nguyen B, Fong C, Luthra A, Smith SA, DiNatale RG, Nandakumar S (2022). Genomic characterization of metastatic patterns from prospective clinical sequencing of 25,000 patients. Cell.

[10] Prasad K, Bloomfield M, Levi H, Keuper K, Bernhard SV, Baudoin NC (2022). Whole-genome duplication shapes the aneuploidy landscape of human cancers. Cancer Res.

[11] Matsumoto T, Wakefield L, Tarlow BD, Grompe M (2020). In vivo lineage tracing of polyploid hepatocytes reveals extensive proliferation during liver regeneration. Cell Stem Cell.

[12] Matsumoto T, Wakefield L, Peters A, Peto M, Spellman P, Grompe M (2021). Proliferative polyploid cells give rise to tumors via ploidy reduction. Nat Commun.

[13] Gentric G, Maillet V, Paradis V, Couton D, L’Hermitte A, Panasyuk G (2015). Oxidative stress promotes pathologic polyploidization in nonalcoholic fatty liver disease. J Clin Investig.

[14] Toyoda H, Bregerie O, Vallet A, Nalpas B, Pivert G, Brechot C (2005). Changes to hepatocyte ploidy and binuclearity profiles during human chronic viral hepatitis. Gut..

[15] Ng IO, Lai EC, Ho JC, Cheung LK, Ng MM, So MK (1994). Flow cytometric analysis of DNA ploidy in hepatocellular carcinoma. Am J Clin Pathol.

[16] Nagasue N, Kohno H, Hayashi T, Yamanoi A, Uchida M, Takemoto Y (1993). Lack of intratumoral heterogeneity in DNA ploidy pattern of hepatocellular carcinoma. Gastroenterology..

[17] Bou-Nader M, Caruso S, Donne R, Celton-Morizur S, Calderaro J, Gentric G (2020). Polyploidy spectrum: a new marker in HCC classification. Gut..

[18] Sigl-Glöckner J, Brecht M (2017). Polyploidy and the cellular and areal diversity of rat cortical layer 5 pyramidal neurons. Cell Rep.

[19] Darzynkiewicz Z. Critical aspects in analysis of cellular DNA content. Curr Protoc Cytom. 2010;7:7.2. 10.1002/0471142956.cy0702s56 PMC3238682 21455968

[20] Tanami S, Ben-Moshe S, Elkayam A, Mayo A, Bahar Halpern K, Itzkovitz S (2017). Dynamic zonation of liver polyploidy. Cell Tissue Res.

[21] Was H, Borkowska A, Olszewska A, Klemba A, Marciniak M, Synowiec A (2022). Polyploidy formation in cancer cells: How a Trojan horse is born. Semin Cancer Biol.

[22] Moein S, Adibi R, da Silva Meirelles L, Nardi NB, Gheisari Y (2020). Cancer regeneration: polyploid cells are the key drivers of tumor progression. Biochim Biophys Acta Rev Cancer.

[23] Zhang S, Mercado-Uribe I, Xing Z, Sun B, Kuang J, Liu J (2014). Generation of cancer stem-like cells through the formation of polyploid giant cancer cells. Oncogene..

[24] Zhang D, Yang X, Yang Z, Fei F, Li S, Qu J (2017). Daughter cells and erythroid cells budding from pgccs and their clinicopathological significances in colorectal cancer. J Cancer.

[25] Zhang L, Yang Z, Zhang S, Zhou K, Zhang W, Ling S (2022). Polyploidy spectrum correlates with immunophenotype and shapes hepatocellular carcinoma recurrence following liver transplantation. J Inflamm Res.

[26] Calderaro J, Ziol M, Paradis V, Zucman-Rossi J (2019). Molecular and histological correlations in liver cancer. J Hepatol.

[27] Llovet JM, Kelley RK, Villanueva A, Singal AG, Pikarsky E, Roayaie S (2021). Hepatocellular carcinoma. Nat Rev Dis Prim.

[28] Rebouissou S, Nault JC (2020). Advances in molecular classification and precision oncology in hepatocellular carcinoma. J Hepatol.

[29] Calderaro J, Couchy G, Imbeaud S, Amaddeo G, Letouzé E, Blanc JF (2017). Histological subtypes of hepatocellular carcinoma are related to gene mutations and molecular tumour classification. J Hepatol.

[30] Miltiadous O, Sia D, Hoshida Y, Fiel MI, Harrington AN, Thung SN (2015). Progenitor cell markers predict outcome of patients with hepatocellular carcinoma beyond Milan criteria undergoing liver transplantation. J Hepatol.

[31] Zeng SS, Yamashita T, Kondo M, Nio K, Hayashi T, Hara Y (2014). The transcription factor SALL4 regulates stemness of EpCAM-positive hepatocellular carcinoma. J Hepatol.

[32] Masuda S, Suzuki K, Izpisua Belmonte JC (2013). Oncofetal gene SALL4 in aggressive hepatocellular carcinoma. N Engl J Med..

[33] Sakaue-Sawano A, Yo M, Komatsu N, Hiratsuka T, Kogure T, Hoshida T (2017). Genetically encoded tools for optical dissection of the mammalian cell cycle. Mol Cell.

[34] Chiang DY, Villanueva A, Hoshida Y, Peix J, Newell P, Minguez B (2008). Focal gains of VEGFA and molecular classification of hepatocellular carcinoma. Cancer Res.

[35] Hao Z, Zhang H, Cowell J (2012). Ubiquitin-conjugating enzyme UBE2C: molecular biology, role in tumorigenesis, and potential as a biomarker. Tumour Biol.

[36] Du R, Huang C, Liu K, Li X, Dong Z (2021). Targeting AURKA in Cancer: molecular mechanisms and opportunities for Cancer therapy. Mol Cancer.

[37] Chen T, Sun Y, Ji P, Kopetz S, Zhang W (2015). Topoisomerase IIα in chromosome instability and personalized cancer therapy. Oncogene..

[38] Nielsen CF, Zhang T, Barisic M, Kalitsis P, Hudson DF (2020). Topoisomerase IIα is essential for maintenance of mitotic chromosome structure. Proc Natl Acad Sci USA.

[39] Sikora E, Czarnecka-Herok J, Bojko A, Sunderland P (2022). Therapy-induced polyploidization and senescence: Coincidence or interconnection?. Semin Cancer Biol.

[40] Carter SL, Cibulskis K, Helman E, McKenna A, Shen H, Zack T (2012). Absolute quantification of somatic DNA alterations in human cancer. Nat Biotechnol.

[41] Boyault S, Rickman DS, de Reyniès A, Balabaud C, Rebouissou S, Jeannot E (2007). Transcriptome classification of HCC is related to gene alterations and to new therapeutic targets. Hepatology..

[42] Steele CD, Abbasi A, Islam SMA, Bowes AL, Khandekar A, Haase K (2022). Signatures of copy number alterations in human cancer. Nature..

[43] Yamashita T, Forgues M, Wang W, Kim JW, Ye Q, Jia H (2008). EpCAM and alpha-fetoprotein expression defines novel prognostic subtypes of hepatocellular carcinoma. Cancer Res.

[44] Chen J, Niu N, Zhang J, Qi L, Shen W, Donkena KV (2019). Polyploid giant cancer cells (PGCCs): the evil roots of cancer. Curr Cancer Drug Targets.

[45] Zhang S, Zhang D, Yang Z, Zhang X (2016). Tumor budding, micropapillary pattern, and polyploidy giant cancer cells in colorectal cancer: current status and future prospects. Stem Cells Int.

[46] Bharadwaj D, Mandal M (2020). Senescence in polyploid giant cancer cells: a road that leads to chemoresistance. Cytokine Growth Factor Rev.

[47] Liu J, Erenpreisa J, Sikora E (2022). Polyploid giant cancer cells: an emerging new field of cancer biology. Semin Cancer Biol.

[48] Niu N, Zhang J, Zhang N, Mercado-Uribe I, Tao F, Han Z (2016). Linking genomic reorganization to tumor initiation via the giant cell cycle. Oncogenesis..

[49] Niu N, Mercado-Uribe I, Liu J (2017). Dedifferentiation into blastomere-like cancer stem cells via formation of polyploid giant cancer cells. Oncogene..

[50] Lin H, Huang YS, Fustin JM, Doi M, Chen H, Lai HH (2021). Hyperpolyploidization of hepatocyte initiates preneoplastic lesion formation in the liver. Nat Commun.

[51] Matsumoto T. Implications of polyploidy and ploidy alterations in hepatocytes in liver injuries and cancers. Int J Mol Sci. 2022;23:9409. 10.3390/ijms23169409 PMC9409051 36012671

[52] Donne R, Saroul-Aïnama M, Cordier P, Celton-Morizur S, Desdouets C (2020). Polyploidy in liver development, homeostasis and disease. Nat Rev Gastroenterol Hepatol.

