# Supplementary material for: Histological diagnosis of polyploidy discriminates an aggressive subset of hepatocellular carcinomas with poor prognosis
Source: Br J Cancer. 2023 Sep 15;129(8):1251–60. doi: 10.1038/s41416-023-02408-6 (PMC10576083; doi:10.1038/s41416-023-02408-6)
Supplement: Supplementary file 1 — Supplementary Text and figures [file 41416_2023_2408_MOESM1_ESM.docx]

Histological diagnosis of polyploidy discriminates an aggressive subset of hepatocellular carcinomas with poor prognosis

**Takanori Matsuura^1,2^,** **Yoshihide Ueda^2^, Yoshiyuki Harada^1,2^, Kazuki Hayashi^1^,**

**Kisara Horisaka^1^, Yoshihiko Yano^2^, Shinichi So^3^, Masahiro Kido^3^, Takumi Fukumoto^3^,**

**Yuzo Kodama^2^, Eiji Hara^1^, Tomonori Matsumoto^1*^**

1. Department of Molecular Microbiology, Research Institute for Microbial Diseases, Osaka University

2. Division of Gastroenterology, Department of Internal Medicine, Kobe University Graduate School of Medicine

3. Division of Hepato-Biliary-Pancreatic Surgery, Department of Surgery, Kobe University Graduate School of Medicine

Supplementary material table of contents:

Figure S1 page 3

Figure S2 page 5

Figure S3 page 6

Figure S4 page 7

Figure S5 page 9

Figure S6 page 10

Figure S7 page 11

Figure S8 Page 12

Figure S9 page 14

Supplemental materials and methods page 15-17

Additional supplemental tables, provided as individual excel tables:

**Supplemental table 1: Genes significantly upregulated in polyploid Huh7-Fucci cells compared to diploids**

It is shown whether genes are upregulated in polyploid pan-cancers (1) and in a proliferation-subtype HCC (2). FC, Fold change; FDR, False discovery rate.

**Supplemental table 2: Genes significantly downregulated in polyploid Huh7-Fucci cells compared to diploids**

FC, Fold change; FDR, False discovery rate.

**Supplemental table 3: Sensitivity and specificity for detecting polyploidy in HCCs**


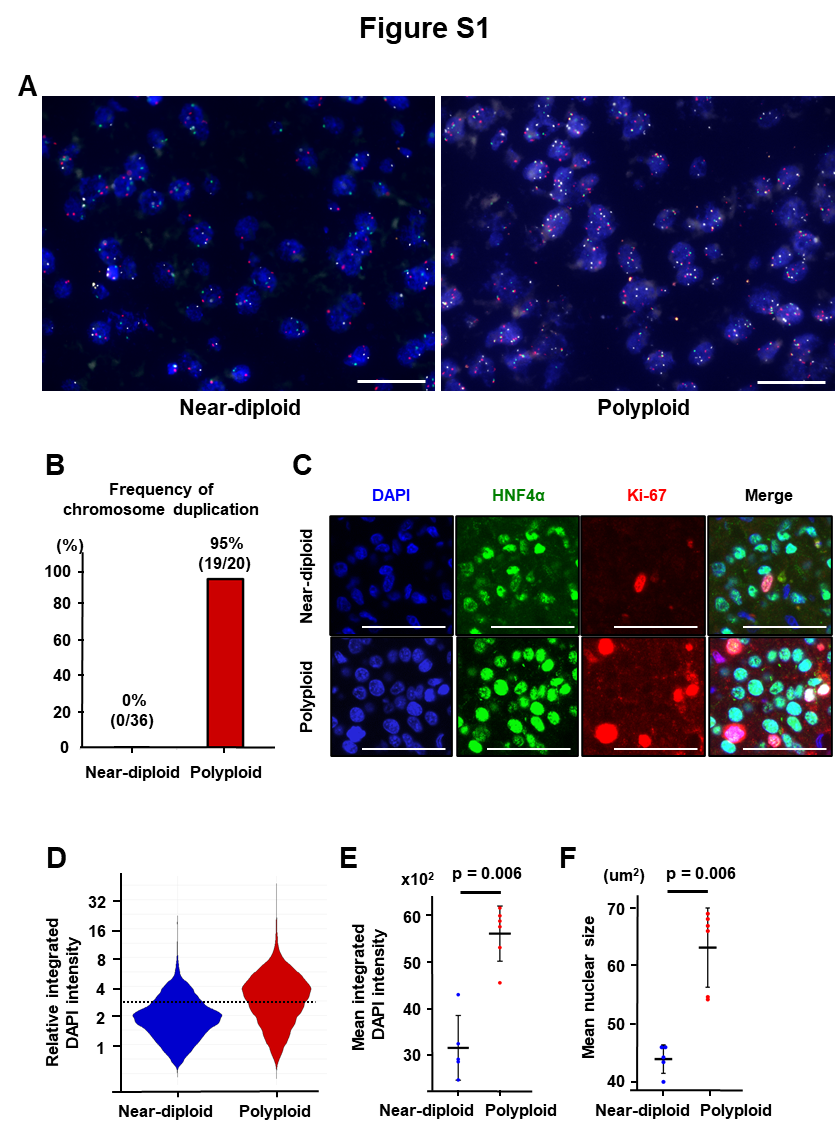


**Figure S1. Evaluation of the ploidy status of HCC**

(A) Wide-filed images of multicolored chromosome FISH. Scale bar, 50μm. (B) Frequency of chromosome duplication. Chromosome duplication (i.e., four or more copies of chromosome) was determined by multicolored chromosome FISH. One polyploid HCC that did not show chromosome duplication had three copies of chromosome 7, 11, and 16. (C) Representative images of HCCs stained with anti-HNF4α, Kι−67 and DAPI. Scale bar, 50 μm. (D) A violin plot of integrated DAPI intensities of each cancer cell nucleus. More than 5153 cells per a tumor on average were analyzed (n = 5 in near-diploid HCC and n = 6 in polyploid HCC), and the violin plot was drawn from the data derived from all cells (31,171 cells in near-diploid and 25,725 cells in polyploid HCCs). A dotted line indicates the border between diploid and polyploid nuclei that was inferred by the modal distribution. (E, F) The integrated DAPI intensities (E) and the size of cancer nuclei (F) in each HCC. The mean value of HNF4α-positive and Ki-67 negative tumor nuclei in each HCC is plotted. Error bars indicate mean ± SD (n = 5 in near-diploid, n = 6 in polyploid, Student’s t-test).


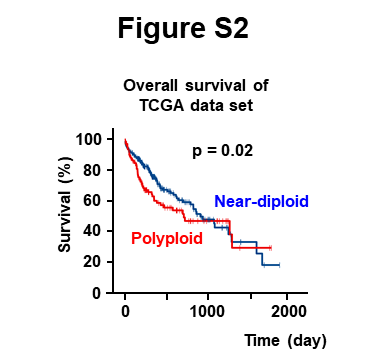


**Figure S2. Kaplan–Meier curve of overall survival in the TCGA data set**

The ploidy status in each case was determined by referring the previous study (3). The p value was calculated by generalized Wilcoxon test.

***
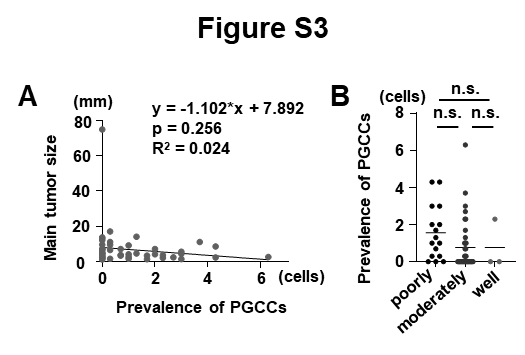
***

**Figure S3. Correlation between the prevalence of PGCCs and tumor size (A) or tumor differentiation (B).**

Linear regression modelling and Mann-Whitney U test were used in (A) and (B), respectively.


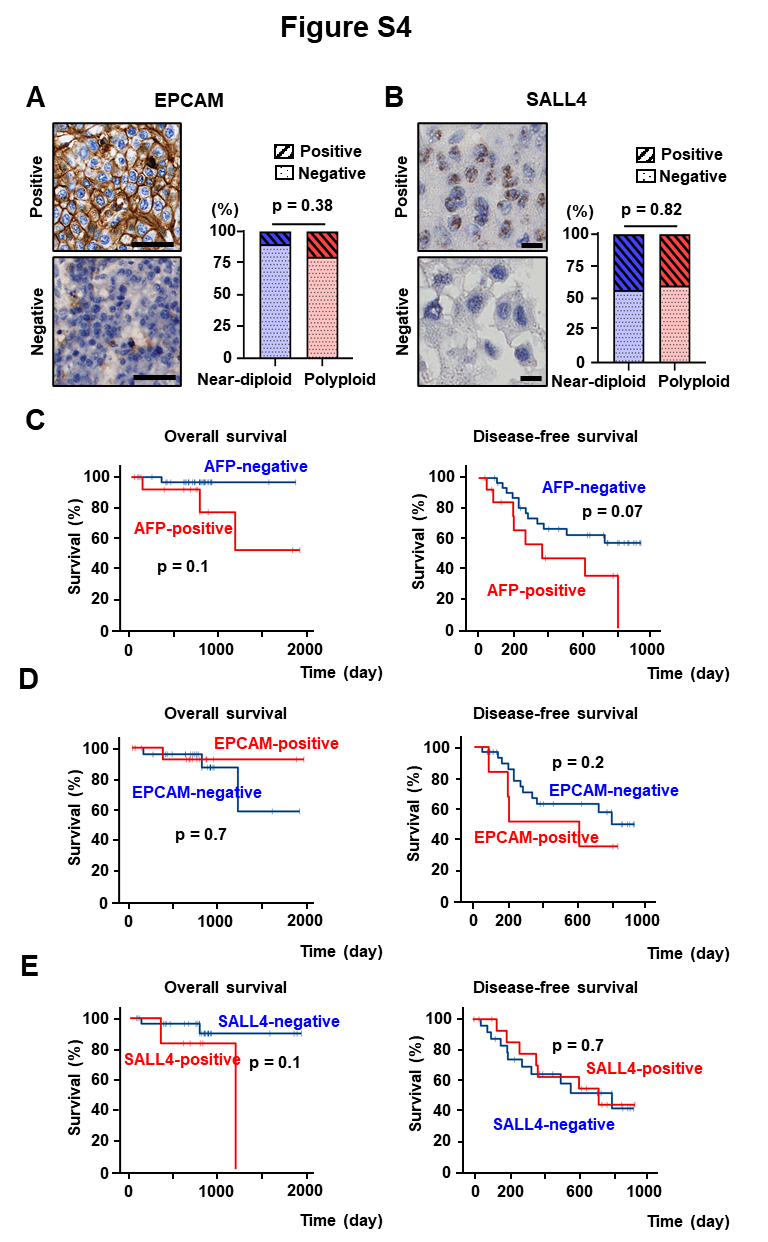


**Figure S4. Correlation between polyploid HCCs and known HCC molecular subtypes.**

(A, B) Immunostaining for EpCAM (A) and SALL4 (B). Scale bar, 50 μm. (C, D, E) Kaplan-Meier curves of overall survival and disease-free survival based on the immunohistochemical positivity of AFP (C), EpCAM (D, and SALL4 (E). The p values were calculated by the generalized Wilcoxon test.


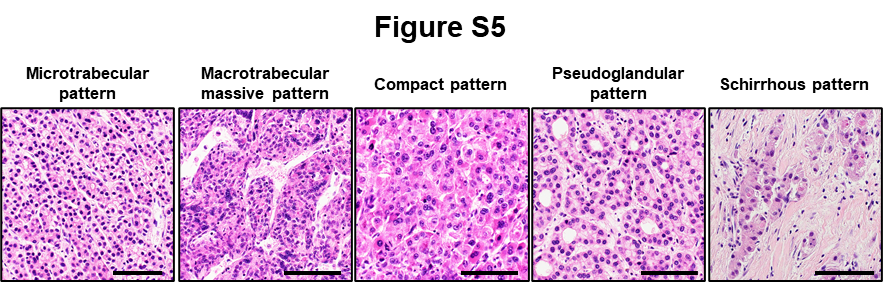


**Figure S5. Architectural growth patterns of HCCs.**

Representative HE images of various architectural growth patterns of HCCs are shown. Scale bar, 50 μm.


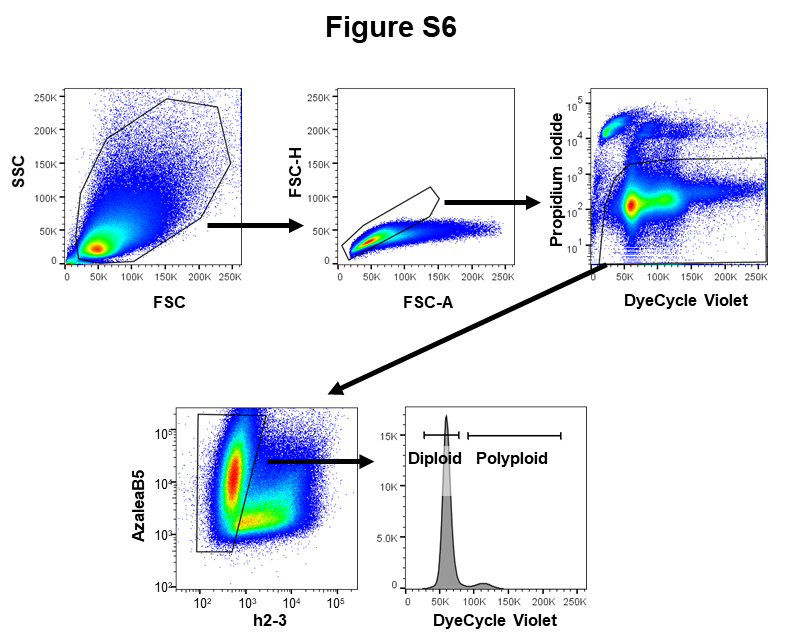


**Figure S6. Sorting strategy for diploid and polyploid Huh7-Fucci cells.**

FSC, forward scatter; SSC, side scatter.


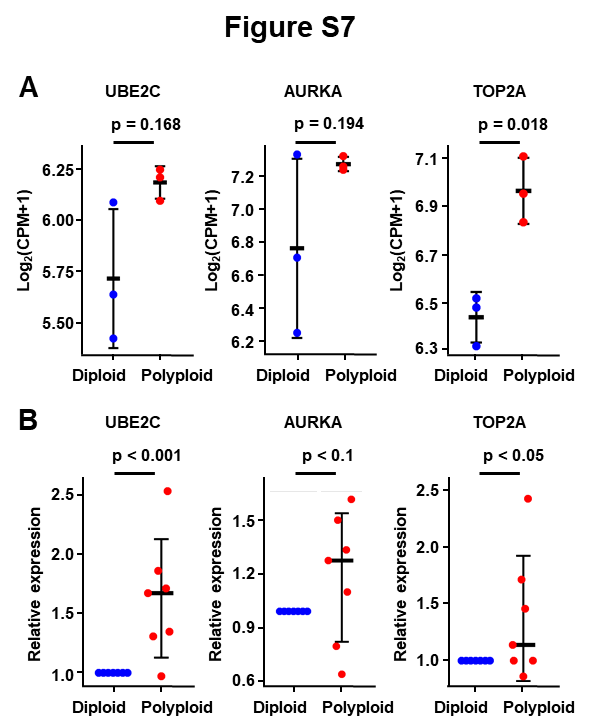


**Figure S7. Transcriptional upregulation of UBE2C, AURKA, and TOP2A genes in polyploid RPE1 cells.**

(A) RNA sequencing data of sorted diploid and polyploid RPE1 cells. A Fucci-expressing cell line was established from human immortalized epithelial RPE1 cells, and RNA sequencing analysis was conducted on diploid and polyploid cells in the G1 phase after collection via FACS. Normalized count data is shown. CPM, count per million. (B) Quantitative reverse transcription-PCR of sorted diploid and polyploid RPE1 cells. The mRNA expression levels of each gene were calculated relative to the housekeeping GAPDH gene. Student’s t-test was performed.


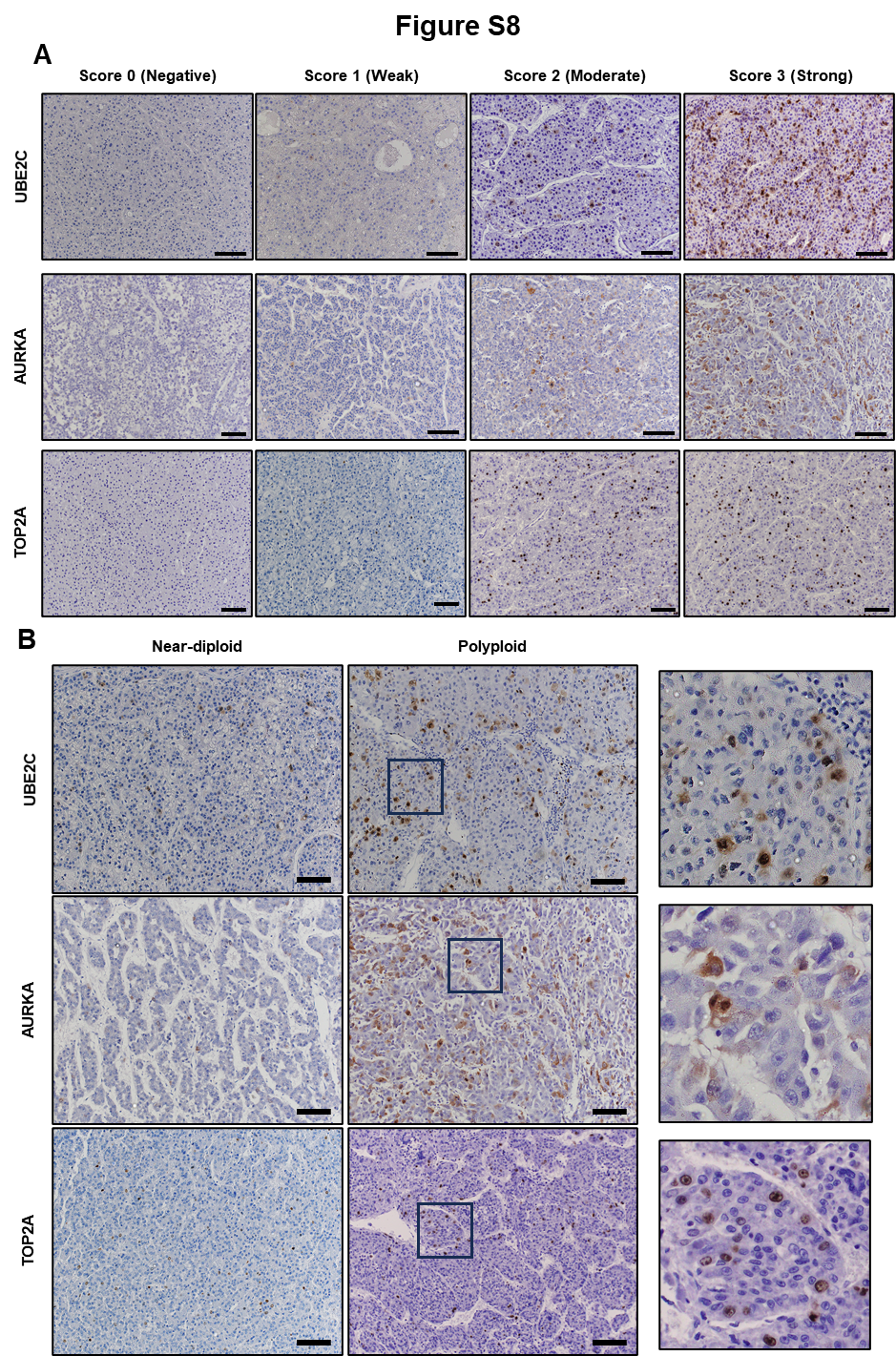


**Figure S8. Immunostaining of UBE2C, AURKA, and TOP2A.**

(A) Scoring criteria of the immunohistostaining in UBE2C, AURKA, and TOP2A. The number of positive cells per field at 200× magnification was calculated and scored as follows; 2 or less: score 0 (negative), 3 to 10 cells: score 1 (weak), 11 to 50 cells: score 2 (moderate), and more than 50 cells: score 3 (strong). Scale bar, 50 μm. (B) Representative wide-field images of immunostaining for UBE2C, AURKA, and TOP2A. High magnification images of polyploid HCCs are also shown in the right panels. Scale bar 200μm.


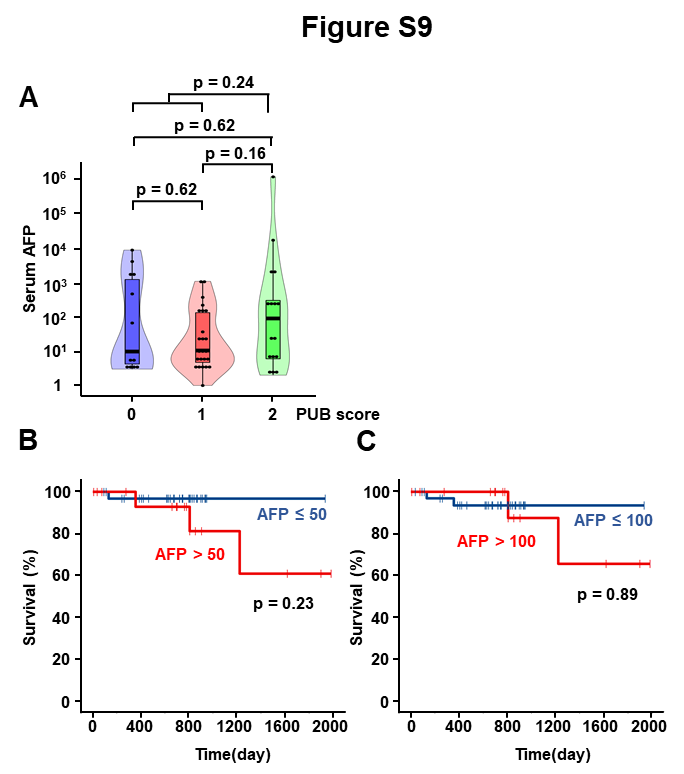


**Figure S9. The significance of serum AFP levels in PUB score and HCC prognosis.**

(A) Plots of serum AFP levels in HCCs with each PUB score. The serum AFP level was slightly but not significantly higher in tumors with a PUB score of 2 than the others. (B, C) Kaplan-Meier curve of overall survival stratified by serum AFP levels.

Supplemental materials and methods

Plasmid constructs and generation of stable cell lines

Huh7 cells were obtained from the Japanese Collection of Research Bioresources (JCRB) and maintained in Dulbecco's modified Eagle's medium supplemented with 10% fetal bovine serum. Huh7-Fucci cells were established using Sleeping Beauty transposon system. A sleeping-beauty transposon plasmid encoding the Fucci cassette (pT3-CAG-tFucci(CA)) was constructed by substituting the Myc gene in pT3-CAG-Myc (4) with the Fucci cassette along with the blasticidin-resistance gene derived from tFucci(CA)5 (Addgene plasmid # 153521 (5)). Huh7 cells were transfected with pT3-CAG-tFucci(CA) and pCMV(CAT)T7-SB100 (Addgene plasmid # 34879 (6)) encoding SB100X transposase, and cells stably expressing Fucci were selected using blasticidin for over a month.

Fluorescence-activated cell sorting (FACS)

Huh7-Fucci cells were harvested from dishes using TrypLE (ThermoFisher Scientific, Waltham, MA) and collected by centrifugation at 200 × g for 5 min. The collected cells were suspended in a phosphate buffer solution containing 2% fetal bovine serum. Cells were incubated with 5 μM Vibrant DyeCycle Violet stain (ThermoFisher Scientific) for 20 min at 37°C to detect ploidy and stained with propidium iodide (Sigma-Aldrich) to exclude dead cells. FACS was performed using BD FACSAria III (Becton Dickinson, Franklin Lakes, NJ, USA). Sorted cells were collected in cooled RNAprotect Cell Reagent (Qiagen, Venlo, The Netherlands) and stored in a deep freezer until processing. FACS data were processed using the FlowJo software (TreeStar).

RNA sequencing analysis

Total RNA was extracted from sorted Huh7-Fucci cells using the RNeasy Micro Kit (Qiagen), following the manufacturer’s instructions. Full-length cDNA was generated using a SMART-Seq HT Kit (Takara Bio), and an Illumina library was prepared using a Nextera DNA Library Preparation Kit (Illumina), following the SMARTer manufacturer’s instructions. Sequencing was performed on an Illumina NovaSeq 6000 sequencer (Illumina) in 100-base paired-end mode at the Genome Information Research Center, Research Institute for Microbial Diseases, Osaka University. The sequencing reads were mapped to the GRCh38 human reference genome (Ensembl release 100) using STAR (version 2.7.4a). The reads per gene were counted using HTSeq (version 0.12.4). Differential gene expression analysis was performed using edgeR (version 3.30.3). Statistical significance was determined using quasi-likelihood F-tests, and the p values were adjusted using the Benjamini–Hochberg false discovery rate (FDR) correction at 0.05. Gene set enrichment analysis was performed by the Broad Institute GSEA (version 4.2.0) using the gene list pre-ranked according to the singed fold change × (−log10 FDR).

Quantitative reverse transcription-PCR

Total RNA was extracted using TRIzol (Thermo Fisher Scientific), and cDNA was synthesized using a PrimeScript RT reagent kit (Takara Bio Inc.). Quantitative PCR was performed on a Thermal Cycler Dice Real-Time System III (Takara Bio Inc.) using TB Green Premix Ex Taq II (Takara Bio Inc.). The mRNA expression levels of each gene were calculated relative to GAPDH expression levels. The PCR primer sequences used are as follows: UBE2C, 5- CGTAAAGGAGCTGAGCCGAG-3
(forward) and 5- TCAGGGAAGGCAGAAATCCC-3 (reverse); AURKA, 5- CAGCTAGAGGCATCATGGACC-3
(forward) and 5- TGCTGAGTCACGAGAACACG-3 (reverse); TOP2A, 5- AAGCCCTCCTGCTACACATT-3
(forward) and 5- ACCGGTAGTGGAGGTGGAAG-3 (reverse); GAPDH, 5- CAACTACATGGTTTACATGTTC-3
(forward) and 5- GCCAGTGGACTCCACGAC-3 (reverse).

References

1. Quinton RJ, DiDomizio A, Vittoria MA, Kotýnková K, Ticas CJ, Patel S, et al. Whole-genome doubling confers unique genetic vulnerabilities on tumour cells. Nature. 2021;590(7846):492-7.

2. Chiang DY, Villanueva A, Hoshida Y, Peix J, Newell P, Minguez B, et al. Focal gains of VEGFA and molecular classification of hepatocellular carcinoma. Cancer Res. 2008;68(16):6779-88.

3. Taylor AM, Shih J, Ha G, Gao GF, Zhang X, Berger AC, et al. Genomic and Functional Approaches to Understanding Cancer Aneuploidy. Cancer Cell. 2018;33(4):676-89.e3.

4. Matsumoto T, Wakefield L, Peters A, Peto M, Spellman P, Grompe M. Proliferative polyploid cells give rise to tumors via ploidy reduction. Nat Commun. 2021;12(1):646.

5. Ryoko A. Two new coral fluorescent proteins of distinct colors for sharp visualization of cell-cycle progression. In: Sakaue-Sawano A, editor. BioRxiv2020.

6. Mátés L, Chuah MK, Belay E, Jerchow B, Manoj N, Acosta-Sanchez A, et al. Molecular evolution of a novel hyperactive Sleeping Beauty transposase enables robust stable gene transfer in vertebrates. Nat Genet. 2009;41(6):753-61.
